# Efficacy of electro-acupuncture and manual acupuncture versus sham acupuncture for knee osteoarthritis: study protocol for a randomised controlled trial

**DOI:** 10.1186/s13063-018-3138-x

**Published:** 2019-01-25

**Authors:** Jian-Feng Tu, Jing-Wen Yang, Lu-Lu Lin, Tian-Qi Wang, Yu-Zheng Du, Zhi-Shun Liu, Hui Hu, Jing-Jie Zhao, Xiao-Gang Yu, Chun-Sheng Jia, Jun Wang, Tong Wang, Ya-Quan Hou, Xuan Zou, Yu Wang, Jia-Kai Shao, Li-Qiong Wang, Zhang-Sheng Yu, Cun-Zhi Liu

**Affiliations:** 1 School of Acupuncture-Moxibustion and Tuina, Beijing University of Chinese Medicine, Fengtai District, Beijing, China; 2 Department of Acupuncture and Moxibustion, First Teaching Hospital of Tianjin University of Traditional Chinese Medicine, Xiqing District, Tianjin, China; 3 Department of Acupuncture and Moxibustion, Guang’an Men Hospital, China Academy of Chinese Medical Sciences, Xicheng District, Beijing, China; 4 Department of Acupuncture and Moxibustion, Dongfang Hospital, Beijing University of Chinese Medicine, Fengtai District, Beijing, China; 5 Department of Traditional Chinese Medicine, Beijing Friendship Hospital, Capital Medical University, Xicheng District, Beijing, China; 6 Department of Acupuncture and Moxibustion, Beijing Hospital of Traditional Chinese and Western Medicine, Haidian District, Beijing, China; 7 Hebei University of Chinese Medicine, Shijiazhuang, Heibei China; 8 Department of Acupuncture and Moxibustion, Dongzhimen Hospital, Beijing University of Chinese Medicine, Dongcheng District, Beijing, China; 9 Department of orthopedics, Institute of Acupuncture and Moxibustion, China Academy Of Chinese Medicine Sciences, Dongcheng District, Beijing, China; 10 Department of Acupuncture and Moxibustion, Beijing Hospital of Traditional Chinese Medicine Affiliated to Capital Medical University, Dongcheng District, Beijing, China; 11 Department of Bioinformatics and Biostatistics, SJTU-Yale Joint Center for Biostatistics, Shanghai Jiao Tong University, Minhang District, Shanghai, China

**Keywords:** Knee osteoarthritis, Electro-acupuncture, Manual acupuncture, Sham acupuncture, Randomised controlled trial

## Abstract

**Background:**

Knee osteoarthritis (KOA) is one of the most common musculoskeletal disorders. Although the available evidence for its efficacy is inconclusive, acupuncture is used as an alternative therapy for KOA. The aim of this trial is to determine the efficacy of electro-acupuncture and manual acupuncture versus sham acupuncture for KOA.

**Methods/design:**

This is a study protocol for a randomised, three-arm, multicentre, clinical trial. A total of 480 patients with KOA will be randomly assigned to the electro-acupuncture group, the manual acupuncture group or the sham acupuncture group in a 1:1:1 ratio. All patients will receive 24 sessions over 8 weeks. Participants will complete the trial by visiting the research centre at week 26 for a follow-up assessment. The primary outcome is the success rate: the proportion of patients achieving a minimal clinically important improvement, which is defined as ≥2 points on the numerical rating scale and ≥6 points on the Western Ontario and McMaster Universities Osteoarthritis Index (WOMAC) function score at week 8 compared with baseline. Secondary outcomes include the numerical rating scale, WOMAC score, global patient assessment and quality of life at weeks 4, 8, 16 and 26 after randomisation.

**Discussion:**

This trial may provide high-quality evidence for the efficacy of acupuncture in the treatment of KOA. The results of this study will be published in peer-reviewed journals.

**Trial registration:**

ClinicalTrials.gov, NCT03274713. Registered on 20 November 2017.

**Electronic supplementary material:**

The online version of this article (10.1186/s13063-018-3138-x) contains supplementary material, which is available to authorized users.

## Background

Knee osteoarthritis (KOA) is one of the most common musculoskeletal diseases [1], and it is a major cause of chronic pain and impaired physical function in older adults [2]. An epidemiological survey showed that the prevalence of KOA according to age class ranged from 2.1% to 10.1% for men and from 1.6 to 14.9% for women in Europe [3]. A survey in China revealed that the overall prevalence of symptomatic KOA was 8.1% and that prevalence increased with age [4]. KOA has become a major public health problem that is associated with an increasing burden on society [5].

The aim of treating KOA is to alleviate pain and to improve function and quality of life. Non-steroidal anti-inflammatory drugs (NSAIDs) commonly used to treat this disorder have various side effects [6, 7]. For patients with end-stage KOA, replacement surgery is often recommended [8]. However, all recent guidelines emphasise the importance of nonpharmacologic therapies [1, 9, 10]. Although acupuncture is increasingly used as a nonpharmacologic therapy in clinical practice [11], the available evidence for its efficacy is inconclusive. A meta-analysis revealed that acupuncture was effective for the treatment of chronic pain, including osteoarthritis [12], whereas a recent high-quality randomised controlled trial suggested that neither laser nor needle acupuncture conferred a benefit over a sham treatment for pain or function in patients older than 50 years with moderate or severe KOA [13].

Acupuncture, like drugs, has a dose–effect relationship [14]. A dose of acupuncture can be measured with the cumulative dose (frequency and total number of sessions), the neurophysiological dose (number of needles, retention time and mode of stimulation), location of needles and treatment timing (before or during disease) [15]. In several trials, the dose of acupuncture was far from adequate [16]. A review revealed that the frequency of treatment showed a clear dose–response relationship with pain outcomes. Another review suggested that the frequency of acupuncture for KOA is usually 3–5 sessions per week in China, whereas the frequency is mostly one session per week in Europe and America [17]. Based on previous clinical experience, we speculate that high-dose acupuncture (24 sessions in 8 weeks) may be an effective therapy for KOA.

Electro-acupuncture (EA) and manual acupuncture (MA) are the most commonly used acupuncture styles in China. Therefore, we designed this multicentre randomised controlled trial to determine the effect of high-dose acupuncture (24 sessions in 8 weeks), compared with sham acupuncture (SA), in alleviating pain and improving function in patients with KOA.

The hypotheses are as follows.

In the first step (confirmatory test for EA versus SA):

- H0: Effect of EA = Effect of SA
- H1: Effect of EA ≠ Effect of SA

In the second step (confirmatory test for MA versus SA):

- H0: Effect of MA = Effect of SA
- H1: Effect of MA ≠ Effect of SA

## Methods/design

### Study design

**Fig. 1:**
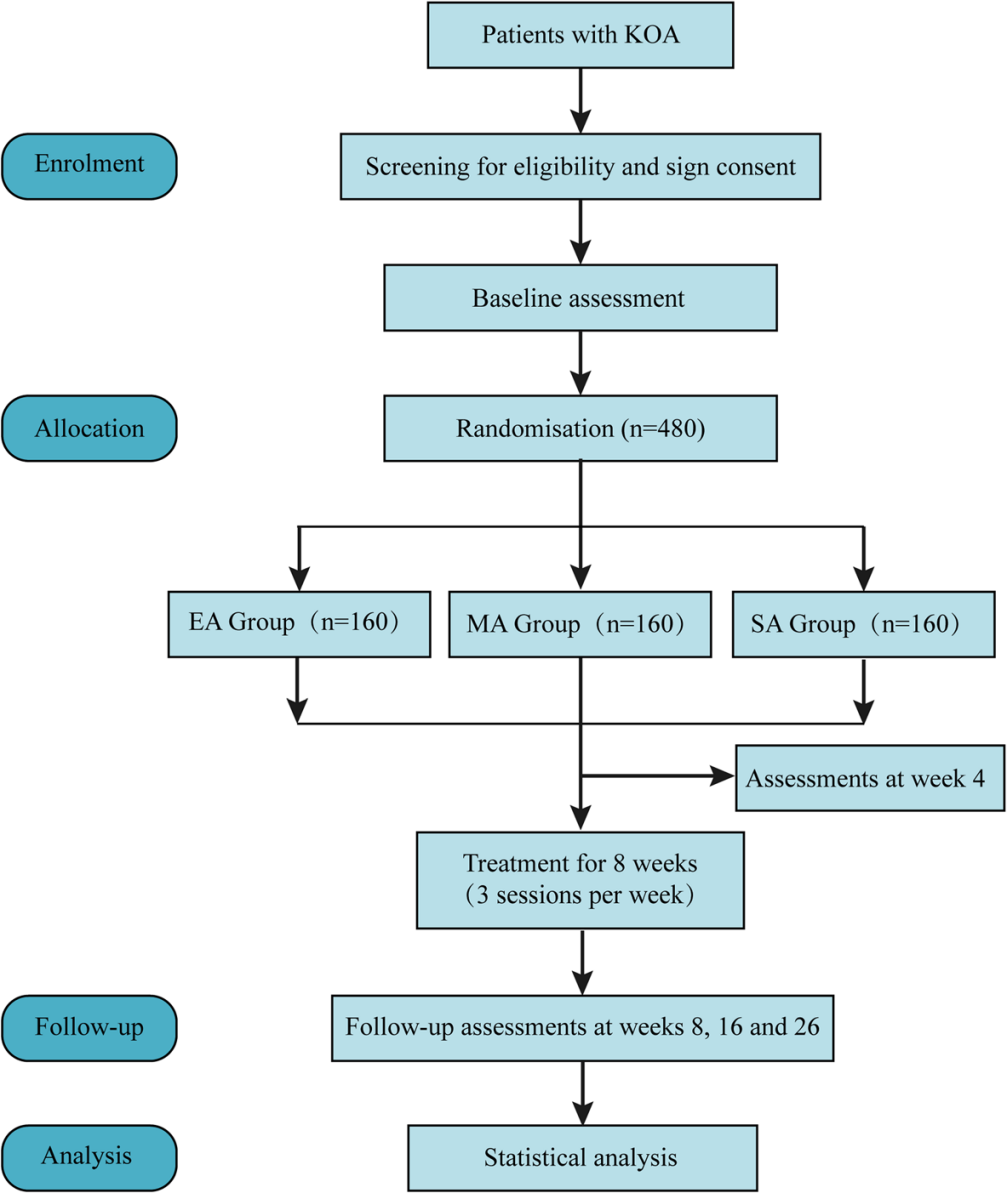
Flow diagram. EA electro-acupuncture, KOA knee osteoarthritis, MA manual acupuncture, SA sham acupuncture

This three-arm, randomised, sham-controlled trial will be conducted at nine hospitals in China: (1) Beijing Hospital of Traditional Chinese Medicine Affiliated to Capital Medical University, (2) Beijing Friendship Hospital Affiliated to Capital Medical University, (3) Dongfang Hospital Affiliated to Beijing University of Chinese Medicine, (4) Dongzhimen Hospital Affiliated to Beijing University of Chinese Medicine, (5) Guang’an Men’s Hospital Affiliated to the China Academy of Chinese Medical Sciences, (6) Hospital of Acupuncture and Moxibustion Affiliated to the China Academy of Chinese Medical Sciences, (7) Beijing Hospital of Traditional Chinese and Western Medicine, (8) First Teaching Hospital of Tianjin University of Traditional Chinese Medicine and (9) the Affiliated Hospital of Hebei University of Chinese Medicine. The study protocol has been approved by the ethics committees at all nine hospitals. The protocol follows the Declaration of Helsinki and will be reported following the SPIRIT guidelines (Additional file 1) [18]. Figure 1 shows the flow diagram of the trial.

### Population and recruitment

**Fig. 2:**
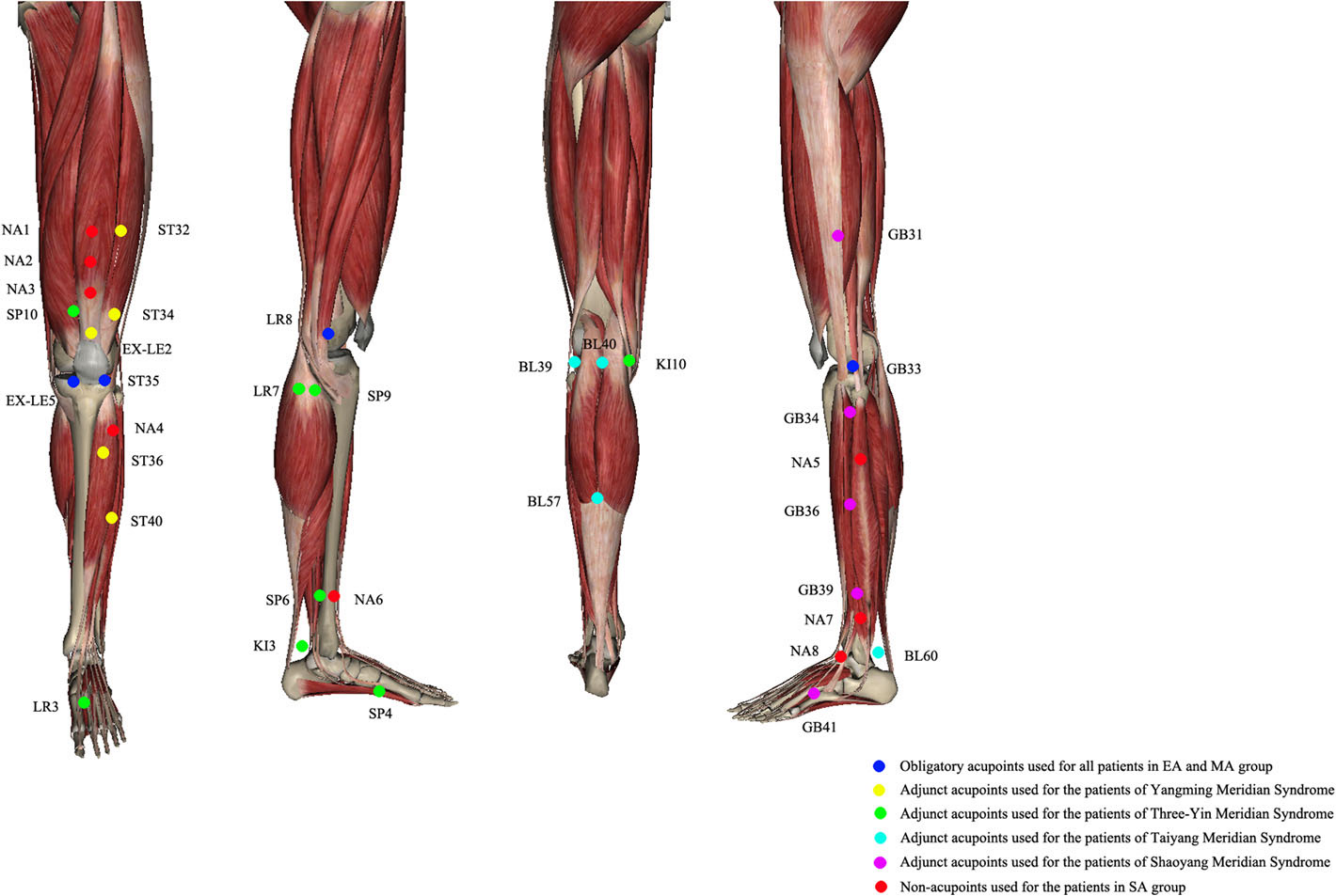
Locations of acupoints and non-acupoints. Dark blue circles: Obligatory acupoints used for all patients in the EA and MA groups. Yellow circles: Adjunct acupoints used for patients with yangming meridian syndrome. Green circles: Adjunct acupoints used for patients with three-yin meridian syndrome. Light blue circles: Adjunct acupoints used for patients with taiyang meridian syndrome. Purple circles: Adjunct acupoints used for patients with shaoyang meridian syndrome. Red circles: Non-acupoints used for patients in the SA group. *EA* electro-acupuncture, *MA* manual acupuncture, *SA* sham acupuncture

KOA patients diagnosed according to the American College of Rheumatology criteria [19] will be recruited through multimodal strategies, including advertisements in hospital social media (WeChat) and newspapers, publicity at community service centres and a clinical patient database. Informed consent will be obtained before randomisation. The schedule of enrolment, intervention and assessments is shown in Fig. 2.

#### Inclusion criteria

1. Aged 45–75 years (either sex)
2. Unilateral or bilateral chronic knee pain for the last 6 months
3. Radiologic confirmation of KOA (Kellgren–Lawrence grade II or III [20])
4. Average pain intensity of 4 or more out of 10 on a numerical rating scale (NRS) in the last week
5. Written informed consent

#### Exclusion criteria

1. History of knee arthroplasty for the most painful knee or waiting for any knee surgery for either knee
2. Knee pain caused by other diseases (such as autoimmune diseases, infection, malignant tumours, trauma, fracture, joint bodies, severe effusion of joint cavity, lumbosacral vertebrae disease, etc.)
3. Arthroscopy in the last 12 months or an intra-articular injection within 6 months
4. Acupuncture treatment in the last 3 months
5. Serious acute or chronic organic diseases or psychiatric disorders
6. Blood coagulation disorders
7. Cardiac pacemaker, metal allergy or needle phobia
8. Pregnant or breastfeeding
9. Participated in other clinical trials in the past 3 months

#### Randomisation and allocation concealment

Eligible patients will be randomly assigned to the EA group, MA group or SA group in a 1:1:1 ratio using a central web-based randomisation tool. The blocked randomisation sequence will be generated with the software SAS 9.3 (SAS Institute, Cary, NC, USA) by an independent statistician (Yang Wang, State Key Laboratory of Translational Cardiovascular Medicine, China), who is not involved in the implementation or statistical analysis of the trial. Randomisation will be stratified within the nine enrolment hospitals with a random block size of 6 or 9. The sequence will be embedded into the software (Beijing Guide Technology Co, Ltd). The clinical research coordinator will input the patient information on a tablet computer and will be given a random number.

### Masking

Patients, outcome assessors and statisticians who perform the statistical analyses will be blinded to group assignment.

### Interventions

Each type of acupuncture treatment will consist of 24 sessions of 30-min duration over 8 weeks (three sessions per week, ideally every other day). Multiple sessions in one day will be prohibited. For patients with bilateral osteoarthritis, both knees will be needled, whereas for patients with unilateral osteoarthritis, the acupuncturist will acupuncture only the affected knee [21]. Only licensed acupuncturists who have at least 5 years of experience with acupuncture will perform the treatment. All acupuncturists will be instructed in standardised operating procedures prior to the start of the study, which consist of the locations of acupoints and non-acupoints, and the manipulation of needles. Single-use sterile needles (length: 25–40 mm, diameter: 0.25 mm; Hwato, Suzhou, China) will be used for acupuncture. Other therapies that may affect symptoms will be prohibited, such as analgesia, NSAIDs, COX-2 inhibitors, opioids, glucosamine, fish oil, hydrotherapy, physical therapy, etc. If the patients need rescue medication, they will be provided with 0.65 g of paracetamol per eight hours (Tylenol, Shanghai Johnson & Johnson Pharmaceuticals, Ltd), except during the 48 h before evaluation.

#### Electro-acupuncture

Acupuncture treatment is semi-standardised [21, 22]. The acupuncture prescription, which was developed from clinical practice and a literature review [23], includes five obligatory acupoints and three adjunct acupoints.

**Fig. 3:**
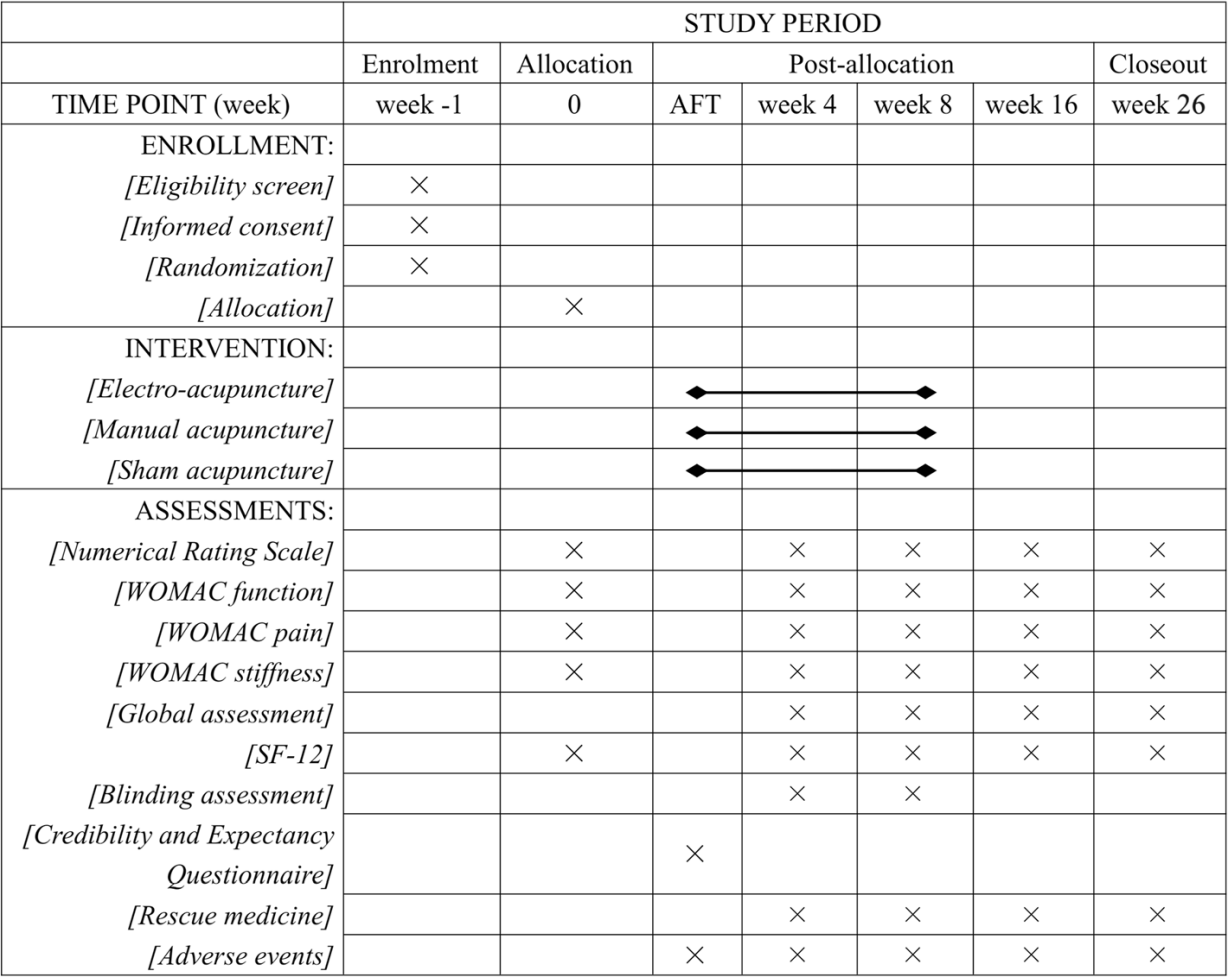
Schedule of enrollment, intervention and assessments (SPIRIT Figure) AFT, after the first treatment; *WOMAC* Western Ontario and McMaster Universities Osteoarthritis Index; *SF-12* 12-item Short Form Health Survey

The obligatory acupoints include *dubi* (ST35), *neixiyan* (EX-LE5), *ququan* (LR8), *xiyangguan* (GB33) and an *ashi* point (the point where the patient feels most pain). Adjunct acupoints will be chosen by the acupuncturists according to traditional Chinese medicine. If pain occurs in the anterior aspect of the affected knee joint, the patient has *yangming* meridian syndrome. Three adjunct acupoints will be chosen from *futu* (ST32), *liangqiu* (ST34), *heding* (EX-LE2), *zusanli* (ST36) and *fenglong* (ST40). If pain occurs in the medial aspect of the affected knee joint, the patient has three-yin meridian syndrome. Three adjunct acupoints will be chosen from *xuehai* (SP10), *yingu* (KI10), *yinlingquan* (SP9), *xiguan* (LR7), *sanyinjiao* (SP6), *taixi* (KI3), *taichong* (LR3) and *gongsun* (SP4). If pain occurs in the posterior aspect of the affected knee joint, the patient has *taiyang* meridian syndrome. Three adjunct acupoints will be chosen from *weiyang* (BL39), *weizhong* (BL40), *chengshan* (BL57) and *kunlun* (BL60). If pain occurs on the lateral aspect of the affected knee joint, the patient has *shaoyang* meridian syndrome. Three adjunct acupoints will be chosen from *fengshi* (GB31), *yanglingquan* (GB34), *waiqiu* (GB36), *xuanzhong* (GB39) and *zulinqi* (GB41). If more than two aspects are affected, three adjunct acupoints will be chosen from those for the relevant syndromes. All acupoints are localised according to the WHO Standard Acupuncture Locations and exhibited in Table 1 and Fig. 3.

**Table 1:** Locations of acupoints for EA and MA groups

	Acupoint	Location
Obligatory acupoints	*Dubi* (ST35)	On the anterior aspect of the knee, in the depression lateral to the patellar ligament
	*Neixiyan* (EX-LE5)	On the anterior aspect of the knee, in the depression medial to the patellar ligament
	*Ququan* (LR8)	On the medial aspect of the knee, in the depression medial to the tendons of the semitendinosus and the semimembranosus muscles, at the medial end of the popliteal crease
	*Xiyangguan* (GB33)	On the lateral aspect of the knee, in the depression between the biceps femoris tendon and the iliotibial band, posterior and proximal to the lateral epicondyle of the femur
	*Ashi* point	The point where the patient feels most pain
Adjunct acupoints for *yangming* meridian syndrome	*Futu* (ST32)	On the anterolateral aspect of the thigh, on the line connecting the lateral end of the base of the patella with the anterior superior iliac spine, 6 cun^a^ superior to the base of the patella
	*Liangqiu* (ST34)	On the anterolateral aspect of the thigh, between the vastus lateralis muscle and the lateral border of the rectus femoris tendon, 2 cun superior to the base of the patella
	*Heding* (EX-LE2)	On the anterior aspect of the thigh, in the depression superior to the base of the patella
	*Zusanli* (ST36)	3 cun directly below ST35, and one finger-breadth lateral to the anterior border of the tibia
	*Fenglong* (ST40)	On the anterolateral aspect of the leg, lateral border of the tibialis anterior muscle, 8 cun superior to the prominence of the lateral malleolus
Adjunct acupoints for three-yin meridian syndrome	*Xuehai* (SP10)	On the anteromedial aspect of the thigh, on the bulge of the vastus medialis muscle, 2 cun superior to the medial end of the base of the patella
	*Yingu* (KI10)	On the posteromedial aspect of the knee, just lateral to the semitendinosus tendon, in the popliteal crease
	*Yinlingquan* (SP9)	On the tibial aspect of the leg, in the depression between the inferior border of the medial condyle of the tibia and the medial border of the tibia
	*Xiguan* (LR7)	On the tibial aspect of the leg, inferior to the medial condyle of the tibia, 1 cun posterior to SP9
	*Sanyinjiao* (SP6)	On the tibial aspect of the leg, posterior to the medial border of the tibia, 3 cun superior to the prominence of the medial malleolus
	*Taixi* (KI3)	On the posteromedial aspect of the ankle, in the depression between the prominence of the medial malleolus and the calcaneal tendon
	*Taichong* (LR3)	In the depression anterior to the junction of the first and second metatarsal bones
	*Gongsun* (SP4)	On the medial aspect of the foot, anteroinferior to the base of the first metatarsal bone, at the border between the red and white flesh
Adjunct acupoints for *taiyang* meridian syndrome	*Weiyang* (BL39)	On the posterolateral aspect of the knee, just medial to the biceps femoris tendon in the popliteal crease
	*Weizhong* (BL40)	On the posterior aspect of the knee, at the midpoint of the popliteal crease
	*Chengshan* (BL57)	On the posterior aspect of the leg, at the connecting point of the calcaneal tendon with the two muscle bellies of the gastrocnemius muscle
	*Kunlun* (BL60)	On the posterolateral aspect of the ankle, in the depression between the prominence of the lateral malleolus and the calcaneal tendon
Adjunct acupoints for *shaoyang* meridian syndrome	*Fengshi* (GB31)	On the lateral aspect of the thigh, in the depression posterior to the iliotibial band where the tip of the middle finger rests, when standing up with the arms hanging alongside the thigh
	*Yanglingquan* (GB34)	On the fibular aspect of the leg, in the depression anterior and distal to the head of the fibula
	*Waiqiu* (GB36)	On the fibular aspect of the leg, anterior to the fibula, 7 cun proximal to the prominence of the lateral malleolus
	*Xuanzhong* (GB39)	On the fibular aspect of the leg, anterior to the fibula, 3 cun proximal to the prominence of the lateral malleolus
	*Zulinqi* (GB41)	On the dorsum of the foot, distal to the junction of the bases of the fourth and fifth metatarsal bones, in the depression lateral to the fifth extensor digitorum longus tendon

^a^1 cun (≈20 mm) is defined as the width of the interphalangeal joint of the patient’s thumb

The needles will be stimulated manually for at least 10 s to achieve *de qi* (a compositional sensation including soreness, numbness, distention and heaviness) and then paired electrodes from an EA apparatus (HANS-200A acupoint nerve stimulator, Nanjing Jisheng Medical Co, Ltd) will be attached to the needle handles at LR8 and GB33 and another two adjunct acupoints by the research assistant. The wave will be set as 2/100 Hz. The electric current will be increased until the needles begin to vibrate slightly.

#### Manual acupuncture

Patients in the MA group will undergo similar procedures as the EA group except that no current will be output from the electrical apparatus, which will have an indicator to show that it has working power [24]. The acupuncture prescription includes five obligatory acupoints and three adjunct acupoints.

#### Sham acupuncture

Eight non-acupoints that are separate from conventional acupoints or meridians will be used for the SA group. The schedule, electrode placements and other treatment settings are the same as for the EA group but with superficial skin penetration (2–3 mm in depth) and no electricity output or needle manipulation for *de qi*. The non-acupoints are shown in Table 2 and Fig. 3.

**Table 2:** Locations of non-acupoints for SA group

Non-acupoint	Location
NA1	On the anterior aspect of the thigh, 6 cun^a^ above the upper edge of the patella (between the spleen and stomach meridian)
NA2	On the anterior aspect of the thigh, 5 cun above the upper edge of the patella (between the spleen and stomach meridian)
NA3	On the anterior aspect of the thigh, 4 cun above the upper edge of the patella (between the spleen and stomach meridian)
NA4	In the middle between GB34 and ST36 (between the gallbladder and bladder meridian)
NA5	3 cun below GB34 (between the gallbladder and bladder meridian)
NA6	2 cun above the medial malleolus (between the liver and spleen meridian)
NA7	2 cun above the lateral malleolus (between the gallbladder and bladder meridian)
NA8	In the middle between GB40 and ST41 (between the gallbladder and bladder meridian)

^a^1 cun (≈20 mm) is defined as the width of the interphalangeal joint of the patient’s thumb

Differences among three acupuncture groups are listed in Table 3.

**Table 3:** Differences among three acupuncture groups

	EA	MA	SA
Points stimulated	Acupoints	Acupoints	Non-acupoints
Number of points	8	8	8
Depth of acupuncture (mm)	≥10	≥10	2–3
Manipulation	Y	Y	N
*De qi*	Y	Y	N
Electrical apparatus	Attached	Attached	Attached
Electric current	Y	N	N
Needle retention (min)	30	30	30
Number of sessions	24	24	24

### Outcomes

For patients with unilateral osteoarthritis, the knee affected will be assessed throughout the entire study. For bilateral osteoarthritis, the knee defined at baseline as most painful will be the one assessed throughout the entire study [21].

#### Primary outcome

The primary outcome will be the success rate [25]: the proportion of patients achieving a minimal clinically important improvement (MCII) [13, 26], which is defined as ≥2 points on the NRS and ≥6 points in the Western Ontario and McMaster Universities Osteoarthritis Index (WOMAC) function score at week 8 compared with baseline.

The primary outcome encompasses valid and reliable pain [27] and function [28] measures for KOA. Average knee pain in the previous week will be assessed using an 11-point NRS ranging from ‘no pain’ (0) to ‘worst pain’ (10) (MCII = 2 units, which is extrapolated from 19.9-mm MCII reported for 100-mm visual analogue scales [26, 29]). Physical function will be assessed using the WOMAC function subscale (Likert version 3.1), scored from 0 to 68 with lower scores indicating better function (MCII = 6 units, which is extrapolated from 9.1-unit MCII reported for standardised 100-unit WOMAC function subscale [26]).

#### Secondary outcomes

##### Success rate at other time points

The success rate will also be measured at weeks 4, 16 and 26 after randomisation.

##### Knee-joint pain

The average pain over the previous week will be assessed using an 11-point NRS [27] with scores ranging from 0 to 10. Knee pain will be also assessed using the WOMAC pain subscale [28], which ranges from 0 to 20. It has five items assessing the severity of pain in five different conditions. Both the NRS and the WOMAC pain subscale will be used at weeks 0, 4, 8, 16, and 26 after randomisation. For both, higher scores indicate worse pain.

##### Knee-joint function

The average function over the previous week will be measured using WOMAC function subscale [28] at weeks 0, 4, 8, 16 and 26 after randomisation. The WOMAC function subscale ranges from 0 to 68 and includes 17 items. Lower scores indicate better physical function.

##### Knee-joint stiffness

The average stiffness over the previous week will be measured using the WOMAC stiffness subscale [28] at weeks 0, 4, 8, 16 and 26 after randomisation. The WOMAC stiffness subscale ranges from 0 to 8 and includes two items. Higher scores indicate more stiffness.

##### Patient global assessment

The patient global assessment [30] has one item. Participants will be asked how their knee symptoms were during the past week. The answers include ‘extremely improved’, ‘slightly improved’, ‘not changed’, ‘slightly aggravated’ and ‘extremely aggravated’. This question will be asked at weeks 4, 8, 16 and 26 after randomisation.

##### Quality of life

Quality of life will be assessed at baseline and at weeks 4, 8, 16 and 26 after randomisation using the 12-item Short Form Health Survey (SF-12) [31], which consists of a mental domain and a physical domain. Each domain ranges from 0 to 100. Higher scores indicate a better quality of life.

##### Blinding assessment

To test whether the participants are blinded successfully, all participants will be asked to guess which kind of acupuncture they received at weeks 4 and 8 after randomisation.

##### Credibility and expectancy

The credibility and expectancy of participants will be measured using the Credibility/Expectancy Questionnaire [32] within 5 min after the first treatment.

##### Rescue medicine

Any use of paracetamol will be ascertained at weeks 4, 8, 16 and 26 after randomisation.

### Adverse events

All adverse events will be recorded throughout the trial by patients, outcome assessors and acupuncturists using a specific questionnaire. Based on the potential relationship between needling and adverse events, adverse events will be categorised by acupuncturists and related specialists as treatment-related or not within 24 h of occurrence. Common treatment-related adverse events include subcutaneous hematoma, continuous post-needling pain, itching at the sites of needle insertion, dizziness, etc.

### Data management

Data will be input into an electronic case report form (eCRF), which will be developed by a third-party corporation (Beijing Guide Technology Co, Ltd) before recruitment begins. A third-party contract research organisation (Beijing QiHuang Medicine Clinical Research Centre) will be responsible for verifying the accuracy of the data. Dynamic management will be carried out to ensure that the data are collected completely, promptly and accurately using a validation function in the eCRF. When the trial is completed, the database will be locked by the data management team, after which the researchers can no longer modify the data.

Both paper files and electronic documents will be preserved for at least 5 years after publication. If readers and reviewers have any questions, they can contact the corresponding author for access to the original data. Patient information will remain anonymous, including name, ID number and telephone number.

Furthermore, an independent Data and Safety Monitoring Board will be established to review and interpret the trial data. The board will review the progress of the trial after 3 months, independently of the investigators, and decide if premature closure of the study is required, based solely on adverse events.

### Quality control

The protocol will be reviewed and revised by experts in acupuncture, rheumatology, orthopaedics, methodology and statistics. A pre-specified standard operating procedure (which includes screening patients, taking X-rays, acupuncture, filling out the eCRF, assessing outcomes and data management) will be used. Online monitoring and on-site monitoring will be adopted in this trial. All modifications of the data can be traced through the eCRF.

### Sample size

Acupuncture is a complex intervention that is different from drugs. If the acupoints in the study were changed, then the efficacy of the acupuncture would be altered. For this reason, we do not use data from the literature to calculate the sample size. Based on previous pilot trials [33] and clinical experience, the success rates of the EA, MA and SA groups are expected to be 70%, 60% and 40%, respectively. A two-sided significance level of 0.025 will avoid the inflation of type I errors. A sample size of 56 patients in each group is estimated to have 80% power to detect significant differences between the EA group and the SA group. A sample size of 128 patients in each group is estimated to have 80% power to detect significant differences between the MA group and the SA group. To compensate for a 20% loss to follow-up, the sample size was increased to 160 patients in each group.

### Statistical analysis

Patients’ baseline characteristics will be summarised by treatment arm. Continuous variables will be described using the mean (standard deviation), or the median (interquartile range) if the normality assumption is violated. One-way analysis of variance (ANOVA) or Kruskal–Wallis one-way ANOVA (if normality is violated) will be used for comparison among the three groups. Categorical variables will be described using the frequency (percentage) and compared using the chi-squared test. All analyses will be performed using SAS 9.3 (Cary, NC).

All efficacy analyses will be performed using the intention-to-treat set, which will consist of all patients who have been randomised and have finished at least one post-baseline measurement. For the primary comparisons, we will calculate the success rates at 8 weeks and compare the EA group and the SA group (and between the MA group and the SA group) using the *Z*-test for proportions. We set α = 0.025 to adjust for the multiple comparison.

For the secondary outcomes, continuous variables with repeated measurement, including WOMAC pain, function and stiffness scores, NRS and quality of life (SF-12), will be compared among the three groups at all follow-up time points using a mixed-effect model with repeated measurement methods. If there is a normality violation in the continuous variables, a transformation will be performed before the test. The adverse event rate will be summarised by group and compared using a chi-squared test (or Fisher’s exact test).

A generalised linear mixed-effect model will be used in the sensitivity analysis. This will be performed using the per-protocol set, which includes only those who complete ≥20 sessions and have no major protocol violations (taking other drugs during the trial, etc.). We will perform a subgroup analysis according to the Kellgren–Lawrence grade.

## Discussion

KOA is one of the most common musculoskeletal diseases and causes considerable financial burden for society. This large trial will evaluate the efficacy of EA and MA versus SA in improving the symptoms of KOA.

This trial meets the methodological demand for adequate randomisation, allocation concealment, and blinding of patients, outcome assessors and statisticians. The ‘dose’ of acupuncture in our trial is intensive. It follows traditional Chinese medicine and is like clinical practice in China. There will be 3 treatment sessions per week in the 8-week treatment phase, giving a total of 24 sessions. The needles will be stimulated manually for at least 10 s at each acupoint and retained in situ for 30 min. Additionally, a suitable control group is critical for a well-designed clinical trial. It is accepted that acupuncture is effective for the treatment of KOA compared with a blank control [12]. However, whether acupuncture is better than SA is controversial. Thus, the control group will receive SA in this trial. Although a completely inert placebo control is not possible in acupuncture trials, superficial insertion at non-acupoints with no electric current is one of the most commonly used approaches for administering sham treatments in acupuncture trials, according to a literature review [34]. Patients who have received acupuncture treatment in the last 3 months and can distinguish SA from EA and MA will not be included. The same control has successfully been used to mask participants in other trials in China [24, 35]. Furthermore, all participants will be asked to guess which treatment they have received to test whether the participants are masked successfully. A further 2 × 2 factorial study might be designed to investigate use of acupoints versus non-acupoints, shallow acupuncture versus deep acupuncture, and their interaction.

Acupuncture alleviates pain by activating a variety of bioactive chemicals through peripheral, spinal and supraspinal mechanisms [36]. Opioids play a central role in acupuncture inhibition of all kinds of pain. Opioids desensitise peripheral nociceptors, decrease pro-inflammatory cytokines in peripheral sites and decrease cytokines in the spinal cord. A preclinical investigation indicated that adenosine mediates the effects of acupuncture [37]. EA of 2/100 Hz increased the release of both endomorphin-2 and dynorphin, whereas EA of (2 + 100) Hz increased the release of dynorphin but not of endomorphin-2 [38]. There is a more potent anti-nociceptive effect induced by 2/100 Hz EA, compared with that of (2 + 100) Hz EA. Thus, we chose 2/100 Hz.

A limitation is that this trial will not have enough power to test the difference between EA and MA. Second, the acupuncturists cannot be blinded due to the nature of the intervention. At the end of this trial, we hope the results will provide more reliable evidence and clarify the value of acupuncture as a treatment for KOA.

### Trial status

This trial is currently recruiting patients.

## Additional file

Additional file 1: Completed Standard Protocol Items: Recommendations for Interventional Trials (SPIRIT) 2013 Checklist. Items addressed in this clinical trial protocol. (DOCX 54 kb)

## Abbreviations

ANOVA: One-way analysis of variance
EA: Electro-acupuncture
eCRF: Electronic case report form
KOA: Knee osteoarthritis
MA: Manual acupuncture
MCII: Minimal clinically important improvement
NSAID: Non-steroidal anti-inflammatory drug
NRS: Numerical rating scale
SA: Sham acupuncture
SF-12: 12-item Short Form Health Survey
WOMAC: Western Ontario and McMaster Universities osteoarthritis index
