# Correction to: Efficacy of electro-acupuncture and manual acupuncture versus sham acupuncture for knee osteoarthritis: study protocol for a randomised controlled trial

**DOI:** 10.1186/s13063-019-3338-z

**Published:** 2019-04-10

**Authors:** Jian-Feng Tu, Jing-Wen Yang, Lu-Lu Lin, Tian-Qi Wang, Yu-Zheng Du, Zhi-Shun Liu, Hui Hu, Jing-Jie Zhao, Xiao-Gang Yu, Chun-Sheng Jia, Jun Wang, Tong Wang, Ya-Quan Hou, Xuan Zou, Yu Wang, Jia-Kai Shao, Li-Qiong Wang, Zhang-Sheng Yu, Cun-Zhi Liu

**Affiliations:** 1 School of Acupuncture-Moxibustion and Tuina, Beijing University of Chinese Medicine, Fengtai District, Beijing, China; 2 Department of Acupuncture and Moxibustion, First Teaching Hospital of Tianjin University of Traditional Chinese Medicine, Xiqing District, Tianjin, China; 3 Department of Acupuncture and Moxibustion, Guang’an Men Hospital, China Academy of Chinese Medical Sciences, Xicheng District, Beijing, China; 4 Department of Acupuncture and Moxibustion, Dongfang Hospital, Beijing University of Chinese Medicine, Fengtai District, Beijing, China; 5 Department of Traditional Chinese Medicine, Beijing Friendship Hospital, Capital Medical University, Xicheng District, Beijing, China; 6 Department of Acupuncture and Moxibustion, Beijing Hospital of Traditional Chinese and Western Medicine, Haidian District, Beijing, China; 7 Hebei University of Chinese Medicine, Shijiazhuang, Heibei China; 8 Department of Acupuncture and Moxibustion, Dongzhimen Hospital, Beijing University of Chinese Medicine, Dongcheng District, Beijing, China; 9 Department of orthopedics, Institute of Acupuncture and Moxibustion, China Academy Of Chinese Medicine Sciences, Dongcheng District, Beijing, China; 10 Department of Acupuncture and Moxibustion, Beijing Hospital of Traditional Chinese Medicine Affiliated to Capital Medical University, Dongcheng District, Beijing, China; 11 Department of Bioinformatics and Biostatistics, SJTU-Yale Joint Center for Biostatistics, Shanghai Jiao Tong University, Minhang District, Shanghai, China

**Correction to: Trials (2019) 20:79**

**https://doi.org/10.1186/s13063-018-3138-x**

After publication of the original article [1], the authors have notified us that the Trial registration number NCT03274713 should be replaced by **NCT03366363** in the Abstract section of the paper.

## References

[1] Tu JF (2019). Efficacy of electro-acupuncture and manual acupuncture versus sham acupuncture for knee osteoarthritis: study protocol for a randomised controlled trial. Trials.

